# BaEV-pseudotyped lentiviral vectors enable stable CAR expression and cytotoxic function in NK cells

**DOI:** 10.1371/journal.pone.0348674

**Published:** 2026-05-21

**Authors:** Minji Park, Yuree Lim, Yujung Jo, Dong-Hyeon Jo, Sang-Ki Kim, Hyun-Young Kim, Seung-Hwan Lee, Mijeong Lee, Duck Cho

**Affiliations:** 1 Department of Health Sciences and Technology, SAIHST, Sungkyunkwan University (SKKU), Seoul, Republic of Korea; 2 Department of Biopharmaceutical Convergence, Sungkyunkwan University (SKKU), Suwon, Republic of Korea; 3 Department of Biochemistry, Microbiology and Immunology, Faculty of Medicine, University of Ottawa, Ottawa, Onatrio, Canada; 4 Department of Companion & Laboratory Animal Science, Kongju National University, Yesan, Republic of Korea; 5 Vaxcell-Bio Therapeutics, Hwasun, Republic of Korea; 6 Department of Laboratory Medicine and Genetics, Samsung Medical Center, Sungkyunkwan University School of Medicine, Seoul, Republic of Korea; 7 Cell and Gene Therapy Institute (CGTI), Samsung Medical Center, Seoul, Republic of Korea; CRCL: Centre de Recherche en Cancerologie de Lyon, FRANCE

## Abstract

Lentiviral vectors (LVs) pseudotyped with either vesicular stomatitis virus glycoprotein (VSV-G) or baboon envelope glycoprotein (BaEV) have been studied for chimeric antigen receptor (CAR) transduction in natural killer (NK) cells. However, the stability of CAR expression and the persistence of vector genomes following transduction remain underexplored. We generated CAR-NK92 cells using either VSV-G- or BaEV-pseudotyped LVs and evaluated CAR expression kinetics, cytotoxic function, and vector DNA persistence over time. Primary expanded NK (eNK) cells were further transduced with BaEV-LVs to assess applicability in primary cells. Although VSV-G-CAR-NK92 cells transiently yielded high surface expression of CAR, this expression rapidly declined. Genomic DNA analysis revealed marked degradation of transfer DNA and reduced integration stability of VSV-G-transduced cells. BaEV-LVs, however, supported more sustained CAR expression by NK92 cells, demonstrating persistent gDNA integration and durable cytotoxicity, which were also replicated in eNK cells. In conclusion, these findings support BaEV-LVs as a preferred platform for CAR-NK cell engineering.

## Introduction

The development of chimeric antigen receptor (CAR)-engineered natural killer (NK) cells has been gaining attention as a promising cancer immunotherapy [1,2]. However, efficient and stable gene delivery into NK cells remains a key technical hurdle [3], as NK cells are relatively resistant to viral transduction using conventional methods. Lentiviral vectors (LVs) are commonly used for CAR delivery, although their efficiency is heavily influenced by the envelope pseudotype used for vector packaging [4,5].

The vesicular stomatitis virus glycoprotein (VSV-G) is widely used for pseudotyping LVs due to its high infectivity in a broad range of cell types and compatibility with large-scale manufacturing. However, VSV-G-pseudotyped LVs exhibit limited efficiency and stability in NK cells, with previous studies reporting lower transgene expression [6,7]. In contrast, baboon envelope glycoprotein (BaEV)-pseudotyped LVs have emerged as a more suitable alternative for NK cell engineering, demonstrating superior transduction efficiency [7–9]. Nevertheless, despite this advantage of BaEV-LVs has been described, the basis for their stability has not been clearly defined. Moreover, the long-term expression and functional maintenance of BaEV-derived CAR-NK cells remain poorly characterized.

To directly compare the stability of CAR expression, we prepared VSV-G- and BaEV-pseudotyped LVs encoding the same CAR construct, and equalized viral input based on quantified physical titers to ensure a fair comparison. Although both vectors showed comparable CAR expression at early time points, CAR expression in VSV-G-LV-transduced NK cells rapidly declined over time, coinciding with loss of vector-derived DNA. In contrast, BaEV-LV-transduced cells maintained stable CAR expression and vector genome retention. Furthermore, IL-15 co-expression synergistically enhanced the persistence and cytotoxic activity of BaEV-derived CAR-NK cells.

These results emphasize that, although VSV-G LVs are known to be advantageous for large-scale production and initial transduction, they may fail to support long-term CAR expression in NK cells. Therefore, careful vector selection is critical to ensure both manufacturing feasibility and durable gene expression for successful CAR-NK cell therapies.

## Materials and methods

### Cell lines

The human cell lines K562 (chronic myeloid leukemia), Nalm-6 (B cell lineage acute lymphoblastic leukemia), and NK92 (NK cell lymphoma) were purchased from American Type Culture Collection (ATCC, Manassas, VA, USA). K562 and Nalm-6 cells were cultured with RPMI 1640 medium (SH30027,01, Cytiva, Logan, UT, USA), completed with 10% heat-inactivated fetal bovine serum (FBS) (16000−44, Thermo Fisher Scientific, Waltham, MA, USA); 100 U/mL penicillin; and 100 μg/mL streptomycin (15140122, Gibco, Grand Island, NY, USA). NK92 cells were cultured with 200 U/mL of IL-2 (200−02, PeproTech, Rocky Hill, NJ, USA) added to MEM-α medium (12571063, Gibco) supplemented with 10% heat-inactivated FBS; 10% heat-inactivated horse serum (16050122, Gibco); 100 U/mL penicillin; 100 μg/mL streptomycin; 0.2 mM myo-inositol (I5125, Sigma-Aldrich, St. Louis, MO, USA); 0.02 mM folic acid (#F7876, Sigma-Aldrich); and 0.1 mM 2-mercaptoethanol (21985023, Gibco). Lenti-X 293T cells were obtained from Takara and cultured with DMEM medium (11950065, Gibco) completed with 10% heat-inactivated FBS, 100 U/mL penicillin, and 100 μg/mL streptomycin. All cells were cultured at 37°C, 5% CO_2_ in a humidified incubator.

### Human primary NK cell expansion

NK cells were expanded via co-culture of isolated peripheral blood mononuclear cells (PBMCs) with 100 Gy gamma-irradiated K562-mbIL-18/-21 cells, as previously described [10,11]. PBMCs were isolated from leukoreduction system (LRS) chambers obtained from the Korean Red Cross Blood Center (Southern Blood Center, Republic of Korea). These LRS chambers are residual products from routine blood donations. This study was approved by the Institutional Review Board of the Samsung Medical Center (IRB No. SMC 2021-09-111), which determined that informed consent was not required for this study. All samples were fully anonymized before use. Samples used in this study were obtained between 18 November 2022 and 12 April 2024. Briefly, healthy adult donor-derived PBMCs were isolated by density-gradient centrifugation with LymphoPrep (1858–6, Progen Biotechnik GmbH, Heidelberg, Germany). On day 0 of expansion, PBMCs and irradiated feeder cells were co-cultured in a 24-well plate with RPMI 1640 medium completed with addition of 4 mmol/L L-glutamine (25030081, Gibco). For the first week, 10 U/mL of recombinant human IL-2 was added. After day 7 of expansion, the concentration of IL-2 was increased to 100 U/mL, and 5 ng/mL of soluble IL-15 (200–15, PeproTech) was added. The medium was replaced every 2–3 days. Expanded NK (eNK) cells were used for viral transduction on day 7 of expansion, and for functional assays on day 14 of expansion.

### CAR construct design and plasmid construction

All CD19 CAR constructs used in this study were second-generation CARs. Two types of constructs were generated: one with only a CD19 CAR construct (αCD19 scFv-41BB-CD3ζ), and another in which an IL-15 sequence was linked via a T2A peptide for co-expression (αCD19 scFv-41BB-CD3ζ-IL15). The hybrid human IL-15 sequence was synthesized by fusing the signal peptide of CD33 (GenBank NM_001082618.2) with the mature peptide IL-15 sequence (GenBank NM_000585.5), as previously reported [12]. All constructs were synthesized by Lugen Sci Co., Ltd. (Bucheon-si, Gyeonggi-do, Republic of Korea). Constructs were cloned into the XbaI and SalI restriction sites of the pCDH-MSCV vector (System Biosciences, Palo Alto, CA, USA) using a DNA Ligation Kit (#6023, Takara Bio, Shiga, Japan).

### Lentivirus production

Lentivirus was produced via transfection of the Lenti-X 293T cell line with transfer plasmid, lentiviral packaging vector, and envelope vectors. Briefly, 1.2x10^6^ Lenti-X 293T cells were plated on 10 cm dishes two days before transfection, and the culture medium was replaced with fresh complete DMEM medium 1 hour before transfection. Then 6 μg of transfer plasmid and 6 μg of pSPAX2 were mixed with 1 μg of BaEV-TR for BaEV virus, or with 1 μg of VSV-G for VSV-G virus, or with 0.5 μg of each BaEV-TR and VSV-G vectors for the “Combo” virus. The plasmid mix was combined with 35 μL of Lipofectamine-2000 in 400 μL of serum-free DMEM medium, and added to the Lenti-X 293T plate. After 72 hours of incubation, the viral supernatant was collected, centrifuged at 500xg for 10 min to pellet cell debris, and filtered using a mixed cellulose ester filter (25AS045AS, ADVANTEC, Tokyo, Japan). For virus concentration, Lenti-con Reagent (LGV-1021B, Lugen Sci Co., Ltd.) was added to the viral supernatant and incubated for 16 hours at 4°C, and then the virus was concentrated by centrifugation at 1600xg for 50 min at 4°C. The virus pellet was resuspended with DMEM medium and stored at −80°C.

### Titration of lentivirus

To measure the viral genome titer of virus stocks, 10 μL of viral pellet was resuspended with 140 μL of DMEM medium, and viral RNA was extracted using the QiAMP Viral RNA Mini Kit (52904, Qiagen, Hilden, Germany) following the manufacturer’s instructions. Subsequently, the number of viral copies was quantified using the Lenti-X qRT-PCR Titration Kit (631235, Takara Bio Inc.), and the titer of virus stock was measured by multiplying the quantified number by the fold dilution of the stock.

### Transduction of NK92 cells and human primary NK cells

To transduce NK cells, untreated 24-well plates were pre-coated with 20 μg/mL of RetroNectin (T100B, Takara Bio Inc.). Then 2.5x10^5^ of NK cells were plated per well with lentivirus at the indicated viral copy number, with 10 μg/mL of vectofusin-1 (130-111-163, Miltenyi Biotec, Bergisch Gladbach, Germany) and 200 U/mL of IL-2 in their cognate complete medium. The plates were centrifuged at 1300xg for 90 min at 32°C and then incubated for 16 hours at 37°C in a 5% CO_2_ incubator. The next day, the entire medium was replaced with fresh complete medium containing 200 U/mL of IL-2.

### Cytotoxicity assay

For the short-term cytotoxicity assay, NK cells were co-cultured with CellTrace™ Violet (C34557, Invitrogen, Waltham, Massachusetts, USA) dye-labeled K562 and Nalm-6 target cells at various effector:target (E:T) ratios (0.5: to 4:1, as indicated in figure). Target cells cultured without the addition of NK cells were used as controls. Cells were seeded in 96-well U-bottom plates, centrifuged at 800 rpm for 1 minute, and incubated for 4 hours at 37°C in a 5% CO_2_ incubator. In assays using cryopreserved NK cells, the NK cells were thawed one day before assay and cultured with cognate complete medium for 24 hours. The lysis of target cells was determined by staining co-cultured cells with 1 μL/well of 1 mg/mL propidium iodide (P.I., P3566, Invitrogen), and analyzed using a FACSVerse instrument (BD Biosciences, San Jose, California, USA). The cytotoxicity of effector cells was calculated as: [dead target cell (sample) – dead target cell (control)]/[100-dead target cell (control)]*100%.

For long-term assay, each NK (effector) cell and target cell were labeled with CellTrace™ Far Red (C34564, Invitrogen) and CellTrace™ CFSE (C34554, Invitrogen, Waltham Massachusetts, USA), respectively, and co-cultured at an E:T ratio of 1:4. After 24, 48 and 72 hours of co-culture, cells were stained with the LIVE/DEAD™ Fixable Violet Dead Cell Stain Kit (L34964, Invitrogen), and live target cell data were collected using the FACSVerse instrument. The percentage of the live target cell population was calculated by gating each effector and target cell. The gating strategy is indicated in S2 Fig.

### Flow cytometry and antibodies

The following antibodies were used for cell staining: anti-CD3 (clone SK7, 47−0036042) and anti-CD56 (clone CMSSB, 25-0567-42) from Invitrogen; anti-FMC63 (clone Y45, FM3-Y45) from ACROBiosystems (Newark, Delaware, USA); and anti-mouse IgG (clone RMG1−1, 406609) from BioLegend (San Diego, California, USA). Flow cytometry data were acquired using either FACSVerse or FACSLyric (BD Biosciences) and analyzed using Kaluza software (Beckman Coulter Inc., Brea, California, USA).

### Quantitative real-time PCR (qRT-PCR)

For analysis of the gene expression level of CD19 scFv, the mRNA level of CD19 scFv and the housekeeping gene human GAPDH were quantified by qRT-PCR. Briefly, NK92 cells transduced with lentivirus were harvested on day 7 and 14 after transduction, pelleted by centrifugation, and cryopreserved for RNA preparation. Total RNA was extracted using the RNeasy Mini Kit (74104, Qiagen) and reverse transcribed into cDNA using the RevertAid First Strand cDNA Synthesis Kit (K1622, Thermo Fisher Scientific). PCR reaction mixtures were prepared by mixing 1 μl (50 ng) of cDNA with 0.5 μl of each primer at 10 μM, 12.5μl of Power SYBR Green PCR Master Mix (4367659, Applied Biosystems, Foster City, CA, USA), and 10.5 μl of deionized water. qRT-PCR was performed using the QuantStudio 6 Flex (4485692, Applied Biosystems), under the following thermal cycling conditions: a hold stage at 95°C for 10 min and then 40 cycles of 95°C for 15 s and 60°C for 1 min. A melting curve analysis was performed for confirmation of qRT-PCR specificity [13,14].

### IL-15 by ELISA

Secretion of IL-15 was quantified with the Human IL-15 ELISA Set (559268, BD Biosciences) following the manufacturer’s instructions. Cell culture supernatants of each CAR-NK cell type were collected at 24 and 48 hours of culture (1x10^6^/mL), without addition of any exogenous cytokine.

### Cell survival and proliferation analysis

To assess the effect of IL-15 secretion on the resilience of NK cells, NK cells were cultured for 7 days under cytokine starvation; 3x10^4^ cells were seeded in 96-well flat-bottom plates, and the living cell number was counted on the indicated day using CountBright absolute counting beads (C36950, Invitrogen) with annexin-V (A35122, Thermo Fisher Scientific) and P.I. staining. The entire medium was refreshed every 2–3 days.

### Statistical analysis

Statistical analyses were performed using the GraphPad Prism 10 software (GraphPad Software, San Diego, CA, USA). Data are presented as mean ± standard deviation (SD). A two-tailed t-test was used to analyze the differences between two groups, and one-way ANOVA with Tukey’s multiple comparisons test was used for more than two groups. Significant differences were determined as *p < 0.05, **p < 0.01, ***p < 0.001, and ****p < 0.0001.

## Results

### Comparison of lentivirus production yield, CAR expression kinetics, and functional persistence between BaEV- and VSV-G-pseudotyped LVs

**Fig 1:**
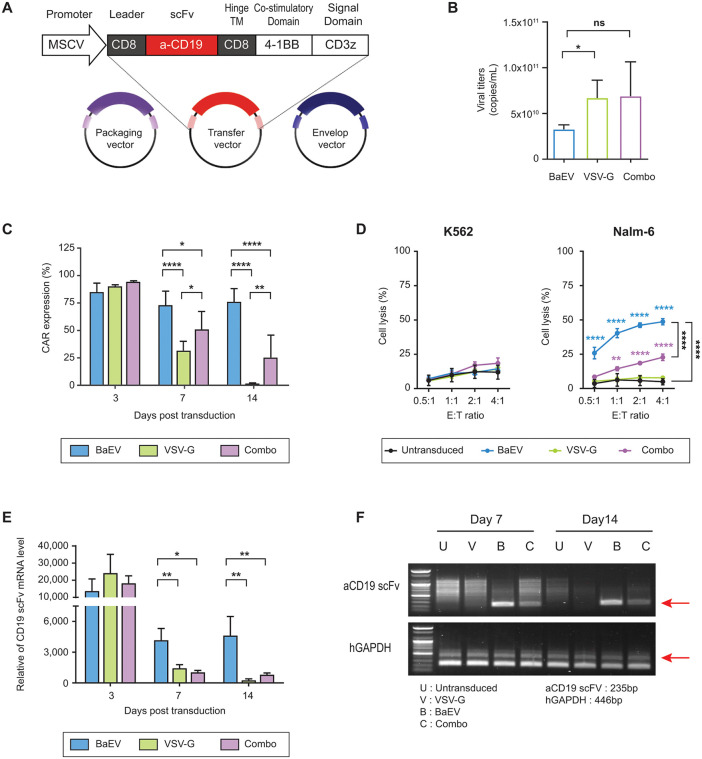
Generation of CAR-NK92 cells using BaEV-, VSV-G- and Combo-LVs, and assessment of CAR expression and CAR-specific cytotoxicity. (A) Schematic representation of the transfer vector used in this study. (B) Average virus titer (viral genome copies/mL) of each LV (n = 5). (C) A total of 2.5x10^5^ NK92 cells were transduced with each type of lentiviral particle using 2.32x10^9^ viral genome copies. The CAR expression level of each CAR-NK92 cell group was assessed by CD19 scFv-positive cell percentage on days 3, 7, and 14 post-transduction (day 3, n = 4; days 7 and 14, n = 5). (D) Cytotoxic activity of untransduced (UTD) and CAR-NK92 cells against K562 and Nalm-6. Cells were co-cultured for 4 hours at indicated E:T ratios (n = 4). (E) qRT-PCR analysis of the relative gene expression of CAR (n = 3). Data are shown as relative CD19 scFv gene expression using human GAPDH as a reference gene with analysis by the 2-ΔΔC_T_ algorithm. (F) Gel electrophoresis analysis of CD19 scFv and human GAPDH amplified from genomic DNA of both UTD and CAR-NK92 cells at days 7 and 14 after transduction. DNA was extracted as total cellular genomic DNA, which may also contain non-integrated residual viral genomes. Data are presented as mean ± standard deviation (SD). Statistical analysis was performed using two-way ANOVA with Tukey’s multiple comparisons test (C), and one-way ANOVA with Tukey’s multiple comparisons test (B, E). *, p < 0.05; **, p < 0.01; ****, p < 0.0001; ns, non-significant.

The plasmid constructs used for lentivirus production, including the packaging plasmid (psPAX2) and transfer plasmid encoding αCD19 CAR (19BBz CAR), are illustrated in Fig 1A. Lentiviral particles were generated and pseudotyped with BaEV, VSV-G, or a 1:1 mixture of VSV-G and BaEV (Combo) envelope glycoproteins. The average virus titers (viral copies/mL) were 3.3 × 10¹⁰ for BaEV-LVs, 6.7 × 10¹⁰ for VSV-G-LVs, and 6.9 × 10¹⁰ for Combo-LVs (Fig 1B). VSV-G and Combo pseudotypes tended to yield two-fold higher titers, whereas BaEV pseudotype exhibited reduced inter-replicate variability.

When cells were transduced with equal viral copy numbers of each type of lentiviral particle, BaEV- and VSV-G-LVs exhibited different transduction efficiency over time. Although the CAR expression was comparable on day 3, only the BaEV-CAR-NK92 cells maintained their CAR expression level over two weeks, whereas the expression by VSV-G-CAR-NK92 significantly declined to 2%. The Combo-LVs showed a similar tendency to that of VSV-G-LVs (Fig 1C). This decline in CAR expression correlated with reduced cytotoxic function. In cytotoxicity assays on day 7 post-transduction, BaEV-CAR-NK92 cells exhibited robust CD19-specific killing (49% lysis at an E:T ratio of 4:1 against Nalm-6 on day 7), whereas cytotoxicity was markedly reduced in VSV-G- and Combo-CAR-NK92 cells in parallel with the loss of CAR expression (Fig 1D).

To determine the cause of this functional and expressional decline, we evaluated post-transduction viability of CAR-NK92 cells and assessed transduction efficiency in HEK293T cells, a conventional model for viral transduction analysis. Cell viability of NK92 cells remained comparable across all groups (S1 Fig. A), and transgene expression in HEK293T cells was stable over time for each LV (S1 Fig. B, C). These results indicate that the loss of CAR expression was not due to vector-induced toxicity or instability of VSV-G. We next evaluated vector persistence by quantifying CAR mRNA levels and vector-derived genomic DNA level. Both mRNA and genomic DNA levels significantly decreased over time in VSV-G- and Combo-CAR-NK92 cells, while BaEV-CAR-NK92 cells retained stable gene expression through day 14 (Fig 1E, F). Taken together, these results highlight that BaEV-LVs enable more durable transduction in NK92 cells, supporting persistent CAR expression and function.

### BaEV-CAR-NK92 cells encoding IL-15 exhibit enhanced proliferation, sustained cytotoxicity, and post-thaw functional stability

**Fig 2:**
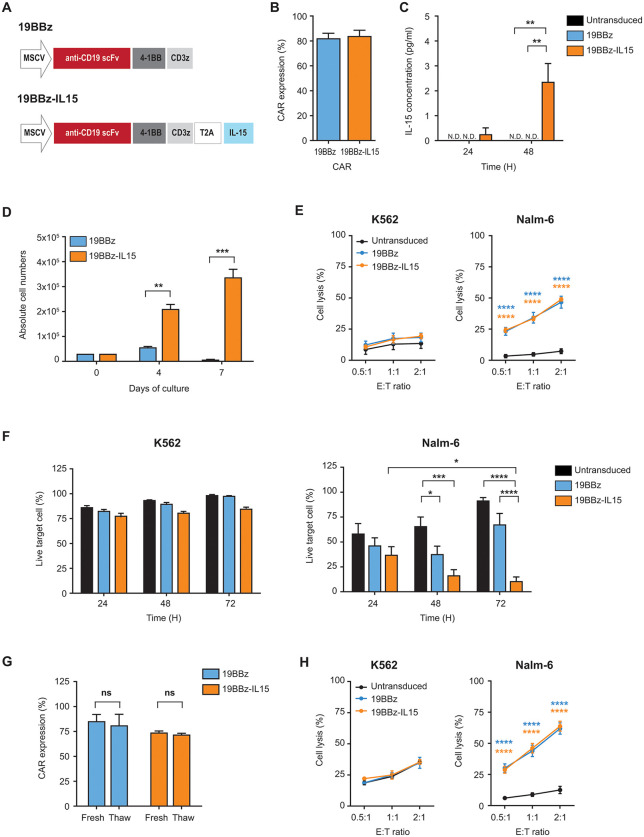
Generation of CAR-IL15-NK92 cells and assessment of CAR expression, CAR-specific cytotoxicity, cell proliferation, and post-thaw CAR stability. (A) Schematic representation of 19BBz and 19BBz-IL15 CAR vectors. (B) CAR expression by NK92 cells transduced with 19BBz CAR or 19BBz-IL15 CAR using BaEV-LVs was measured on day 7 post-transduction (n = 6). (C) IL-15 secretion by both UTD and CAR-NK92 cells cultured without exogenous cytokine for 24 and 48 hours, measured by ELISA (n = 4). (D) Cell survival analysis of each CAR-NK92 cell group performed under cytokine starvation condition. The absolute cell numbers were counted on days 0, 4, and 7 of culture using counting beads with P.I. staining (n = 3). (E) Cytotoxic activity of UTD and CAR-NK92 cells against CD19-negative K562 and CD19-positive Nalm-6 cells. Cells were co-cultured for 4 hours at indicated E:T ratios (UTD, n = 4; CAR, n = 5). (F) Long-term cytotoxicity assay of UTD and CAR-NK92 cells against K562 (left) and Nalm-6 (right) cells. Cells were co-cultured for 24, 48 and 72 hours at an E:T ratio of 0.25:1. The live target cell percentage was calculated by gating each effector and target cell type (K562, n = 3; Nalm-6, n = 6). (G, H) Cryopreserved NK92 cells were thawed and cultured overnight with 200 U/ml of IL-2. At least 70% cell viability was confirmed prior to post-thaw experiments. (G) CAR expression by each CAR-NK92 cell group detected before (Fresh) and after (Thaw) cryopreservation (n = 3). (H) Post-thaw cytotoxic activity of UTD and CAR NK-92 cells against K562 and Nalm-6. Cells were co-cultured for 4 hours at indicated E:T ratios (n = 4). Data are presented as mean ± SD. Statistical analysis was performed using a one-way ANOVA with Tukey’s multiple comparisons test (C), two-way ANOVA with Tukey’s multiple comparisons test (F), and a two-tailed unpaired Student’s t-test (D,G); *, p < 0.05; **, p < 0.01; ***, p < 0.001; ****, p < 0.0001; ns, non-significant.

Given the superior durability of CAR expression and function of BaEV- LVs compared to VSV-G-LVs, we selected BaEV-LVs for subsequent experiments. To promote prolonged survival and steady proliferation of CAR-NK cells, we constructed a CAR vector encoding the IL-15 gene (Fig 2A) and evaluated the transduction efficiency. Using either the 19BBz CAR or 19BBz-IL15 CAR vector, both CAR-NK92 cell types displayed comparable transduction efficiencies (82–84%; Fig 2B). Secretion of IL-15 was detected only in 19BBz-IL15 CAR-NK92 cells (Fig 2C).

To evaluate the effects of IL-15, the proliferation and cytotoxicity of CAR-NK92 cells were assessed. The 19BBz-IL15 CAR-NK92 cells showed prolonged survival and steady proliferation without IL-2, maintaining over 50% viability at day 7 (Fig 2D). In short-term assays, both CAR-NK92 types exhibited similar CAR-specific killing of Nalm-6 targets (Fig 2E). In contrast, long-term assays revealed that IL-15 secretion significantly improved tumor control, with persistent target killing by 19BBz-IL15 CAR-NK92 cells (10% of live target cells at 72 h) (Fig 2F). These findings indicate that BaEV-LV transduction with an IL-15–encoding CAR vector generates CAR-NK92 cells with enhanced proliferation and sustained anti-tumor activity.

We further examined the stability of CAR expression and function after cryopreservation of NK92 cells. Notably, both 19BBz and 19BBz-IL15 CAR-NK92 cells exhibited CAR expression levels comparable to those of fresh cells (Fig 2G) and demonstrated CAR-mediated target cell lysis post-thaw after one day of culture in complete medium (Fig 2H).

### IL-15–encoding BaEV-CAR-eNK cells achieve long-term cytotoxicity and maintain post-thaw activity

**Fig 3:**
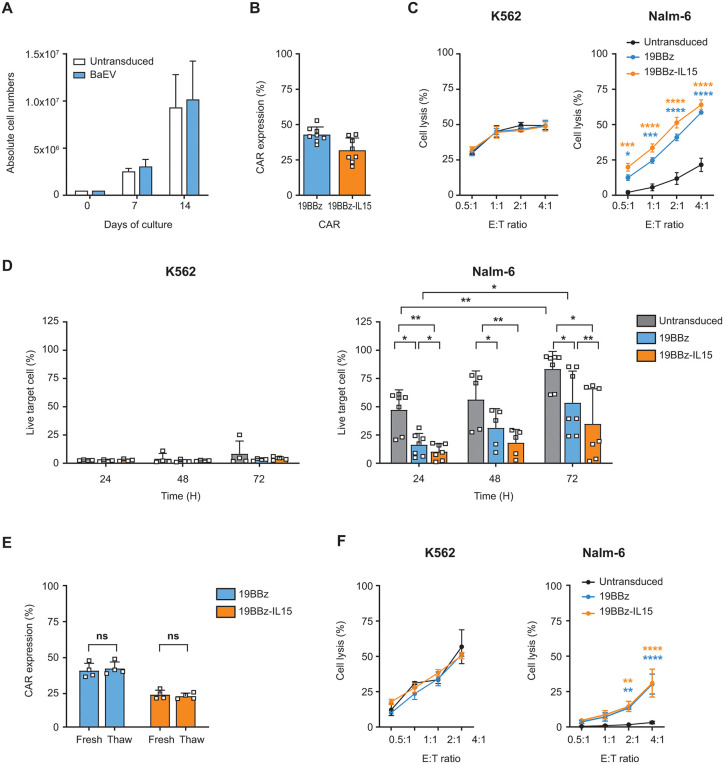
Generation of CAR-IL15 primary NK cells and assessment of CAR expression, CAR-specific cytotoxicity, and post-thaw CAR stability. (A) Cell proliferation of eNK cells transduced with 19BBz CAR using BaEV-LVs. A total of 2.5x10^5^ eNK cells were transduced with each type of lentiviral particle using 1.16x10^9^ viral genome copies (n = 4). (B) CAR expression by eNK cells transduced with 19BBz CAR or 19BBz-IL15 CAR using BaEV-LVs was measured on day 7 post-transduction (n = 8). Each dot represents different donors or replicate experiments for the same donor. (C) Cytotoxicity of UTD and CAR-eNK cells against CD19-negative K562 and CD19-positive Nalm-6 cells. Cells were co-cultured for 4 hours at indicated E:T ratios (n = 3). Cells were used after 7 days of transduction. (D) Long-term cytotoxicity assay of UTD and CAR-eNK cells against K562 (left) and Nalm-6 (right) cells. Cells were co-cultured for 24, 48 and 72 hours at an E:T ratio of 0.25:1. The live target cell percentage was calculated by gating each effector and target cell type (n = 7). Each dot represents different donors or replicate experiments for the same donor. (E, F) Cryopreserved eNK cells were thawed and cultured overnight with 200 U/ml of IL-2. At least 70% cell viability was confirmed prior to post-thaw experiments. (E) CAR expression by each CAR-eNK cell group detected before (Fresh) and after (Thaw) cryopreservation (n = 4). (F) Post-thaw cytotoxic activity of UTD and CAR-eNK cells against K562 and Nalm-6 cells. Cells were co-cultured for 4 hours at indicated E:T ratios (n = 3). Data are presented as mean ± SD. Statistical analysis was performed using a two-way ANOVA with Tukey’s multiple comparisons test (D) and two-tailed unpaired Student’s t-test (E); *, p < 0.05; **, p < 0.01; ns, non-significant.

Given the increasing clinical use of CAR-eNK cells, we next evaluated the BaEV-pseudotyped CAR constructs in ex vivo-expanded primary NK cells from healthy donors. We assessed whether the sustained CAR expression and cytotoxic function observed in NK92 cells could be reproduced in this model. Transduction of eNK cells with BaEV-LVs encoding the 19BBz CAR did not impair their proliferation when compared to UTD eNK cells (Fig 3A). The CAR expression by eNK cells was about 43% (35−52%) for 19BBz CAR and 32% (20−43%) for 19BBz-IL15 CAR, which were maintained for at least 14 days (Fig 3B). The short-term cytotoxicity assay showed similar Nalm-6 target cell lysis rates between both CAR-eNK cell groups (Fig 3C). However, 19BBz-IL15 CAR-eNK cells achieved long-term control of both CD19-negative K562 and CD19-positive Nalm-6 targets over 72 hours. The near-complete loss of K562 cells reflected their high NK cell susceptibility, while improved Nalm-6 clearance highlighted the role of IL-15 in supporting eNK cell survival under cytokine starvation (Fig 3D). After cryopreservation, CAR-eNK cells retained CAR expression levels comparable to those of fresh cells (Fig 3E) and preserved CAR-mediated cytotoxicity, although overall activity was moderately reduced when compared to fresh NK cells (from 21% to 3% in UTD against Nalm-6 at an E:T ratio of 4:1) (Fig 3F).

## Discussion

In this study, we observed that the lentiviral pseudotype significantly influenced the stability and efficiency of CAR delivery in NK cells, with BaEV-pseudotyped vectors supporting more durable expression than VSV-G LVs. In the CAR-NK cell engineering, gene delivery can be achieved through electroporation-based transfection or retroviral/lentiviral vector-mediated transduction. The transfection method enables transient gene expression, whereas viral vectors allow stable genomic integration; in particular, lentiviral transduction is preferred for its ability to transduce non-dividing cells with improved safety features [15]. However, the efficiency of viral transduction depends on the envelope glycoprotein used for pseudotyping, which differs in fusion efficiency, intracellular trafficking, capsid uncoating or receptor usage that may further influence proviral stability and transcriptional competence after entry [8,16–18]. In NK cells, VSV-G-pseudotyped LVs have been reported to exhibit low transduction efficiency, possibly attributed to lack of low-density lipoprotein receptor (LDL-R) expression [6,7]. In contrast, BaEV-pseudotyped LVs have improved transduction efficiency in NK cells, as BaEV envelope utilizes the amino acid transporters ASCT1/2, which are highly expressed on NK cells [6,7,19].

Consistent with their distinct receptor usage, our comparative analyses demonstrated that VSV-G CAR-NK-92 cells only transiently exhibited high CAR expression, which declined rapidly due to limited vector stability and inefficient genomic integration. In contrast, BaEV-LVs supported sustained CAR expression in NK92 cells, with clear evidence of stable genomic integration and durable cytotoxic function for at least two weeks. Together, these results provide direct molecular and functional evidence for the superior transduction durability conferred by BaEV-LVs, emphasizing their strong applicability for stable NK cell engineering.

To further validate the genomic stability and expand the utility of BaEV-LVs, we incorporated the IL-15 sequence in a CAR construct. IL-15 has a well-established role in supporting NK cell survival, proliferation, and functional persistence [20–22]. Consistent with this role of IL-15, the IL-15–encoding BaEV-CAR-NK92 cells demonstrated enhanced proliferation under cytokine deprivation, and sustained effective cytotoxicity in long-term assays. Building on the rising clinical use of ex vivo–expanded NK cells [23,24], we applied the same approach to primary NK cells. BaEV-CAR-eNK cells showed comparable transduction efficiencies across CAR constructs, CAR-dependent cytotoxicity, and IL-15–mediated improvements in long-term tumor control, paralleling the NK92 findings. These results highlight the applicability of BaEV-LVs across CAR constructs and NK cell sources. Notably, CAR expression and CAR-mediated cytotoxicity were preserved following cryopreservation of CAR-NK cells, further confirming the stable transgene expression conferred by BaEV-LVs.

Our results confirm that BaEV pseudotyping enables stable transgene integration and expression in NK cells. Nevertheless, several points warrant discussion. First, the gDNA data used to assess BaEV-mediated stability require careful interpretation. VSV-G-LVs induced substantial early CAR protein and mRNA expression in NK cells, despite poor genomic DNA integration. This finding likely reflects transient expression from residual episomal viral DNA, a phenomenon described for integrase-deficient or poorly integrating LVs, where circular episomal DNA can transiently support transcription before degradation or dilution during NK-cell proliferation [25,26]. Consequently, our data indicate that short-term expression can occur with VSV-G-LVs, but durable CAR expression requires efficient genomic integration, achieved by BaEV-LVs.

Second, regarding the Combo condition (1:1 VSV-G/BaEV), the Combo-LVs showed an intermediate CAR-expression pattern, but this expression still declined over time. Whether this result reflects a stabilizing effect from BaEV or is simply the consequence of co-expressed envelope mixtures remains unresolved, as our current data do not directly demonstrate that the persistence improvement is solely BaEV-driven. Additional experiments are needed to dissect the individual contributions of each envelope. Approaches such as pseudotype-specific barcoding could allow single-cell lineage tracking of BaEV- versus VSV-G–derived transduction events [27]. In parallel, receptor-blocking assays to define envelope–receptor dependencies in NK cells, may further validate these findings [28]. Together, these strategies would clarify how the combined pseudotype affects viral stability and transgene persistence.

Finally, to advance the clinical translation of BaEV-based CAR-NK cells, further optimization and validation are required. Although BaEV pseudotyping markedly improved the stability of CAR expression, transduction efficiency in primary NK cells remains modest. Optimization could involve refined spinoculation parameters, transduction enhancers, or cytokine-based pre-activation protocols to boost gene transfer without compromising viability [5,29]. Furthermore, in vivo evaluation of CAR persistence and antitumor efficacy is needed to confirm the long-term functionality. Such studies, particularly in xenograft models, will be essential to establish the therapeutic potential of BaEV-LV-transduced CAR-NK cells for off-the-shelf clinical applications.

In summary, while VSV-G pseudotyping offers clear manufacturing advantages, it is unsuitable for maintaining long-term CAR expression in NK cells, even at high viral copy input. BaEV pseudotyping consistently supports durable transgene maintenance and functional persistence in both NK92 and primary eNK cells, with added benefits from IL-15 co-expression. These findings emphasize the importance of careful pseudotype selection for CAR-NK manufacturing and support further optimization of BaEV-LV production and cryopreservation protocols to maximize their utility in clinical applications.

## Supporting information

S1 Fig NK92 cell viability post-transduction. (A) Cell viability evaluated on days 3 and 7 after transduction. The percentages of live, early apoptotic and dead cells were assessed using annexin-V and P.I. staining (n = 3). (B) HEK-293T cells were transduced with each type of LVs. The CD19 scFv-positive cell percentage (left) and GFP-positive cell percentage (right) of each group was assessed on days 3 and 7 post-transduction (n = 3) using flow cytometry analysis. (C) Representative dot plots of CD19 scFv (up) and GFP (down) expressions. Data are presented as mean ± SD. Statistical analysis was performed using a two-way ANOVA with Tukey’s multiple comparisons test (B): ns, non-significant.
(PDF)

S2 Fig Flow cytometry gating strategy for cytotoxicity assay. (A, B) Representative gating strategy for (A) short-term killing assay and (B) long-term killing assay.
(PDF)

S3 Fig Transduction of eNK cells using BaEV- and VSV-G-LVs, and BaEV-LV-mediated transduction of NK92 and eNK cells with various viral copy numbers. (A) eNK cells were transduced with each type of lentiviral particle. The CAR expression level of each CAR-eNK cell group was assessed by CD19 scFv-positive cell percentage on days 7 and 14 post-transduction (n = 4). (B) CAR expression by NK92 cells and eNK cells transduced with 19BBz CAR using BaEV-LV at indicated viral copy numbers (NK92, n = 3; eNK, n = 7), (0.01x viral copies, 2.32x10^7^; 0.1x viral copies, 2.32x10^8^; 0.5x viral copies, 1.16x10^9^; 1x viral copies, 2.32x10^9^; transduced into a total of 2.5x10^5^ cells). Data are presented as mean ± SD. Statistical analysis was performed using a two-way ANOVA with Tukey’s multiple comparisons test (A): **, p < 0.01; ****, p < 0.0001; ns, non-significant.
(PDF)

S4 Fig Raw images of Fig 1F gel results. (PDF)
