# Supplementary figures and images for: BaEV-pseudotyped lentiviral vectors enable stable CAR expression and cytotoxic function in NK cells
Source: PLoS One. 2026 May 21;21(5):e0348674. doi: 10.1371/journal.pone.0348674 (PMC13193545; doi:10.1371/journal.pone.0348674)

**A**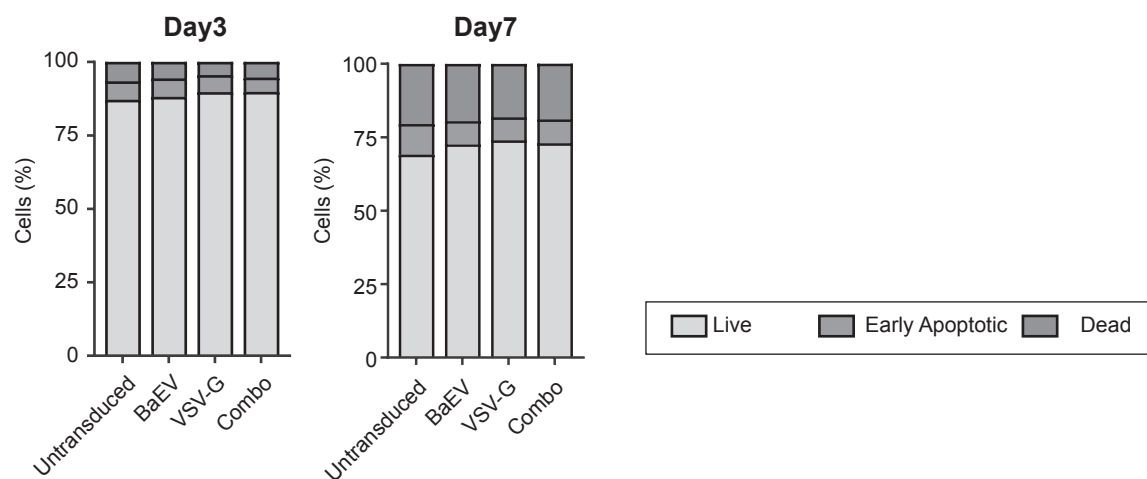**B**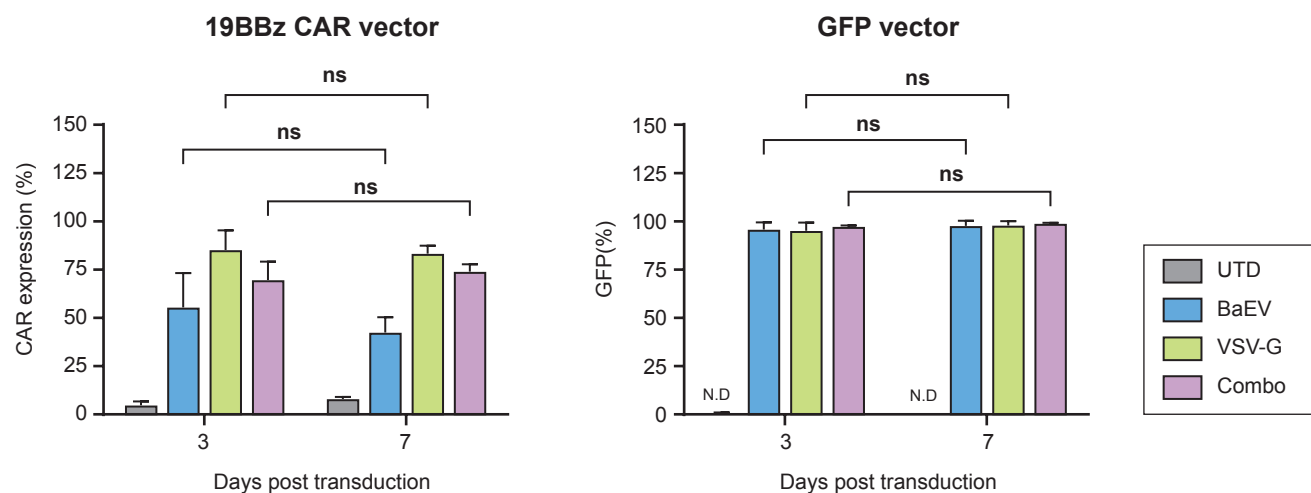**C**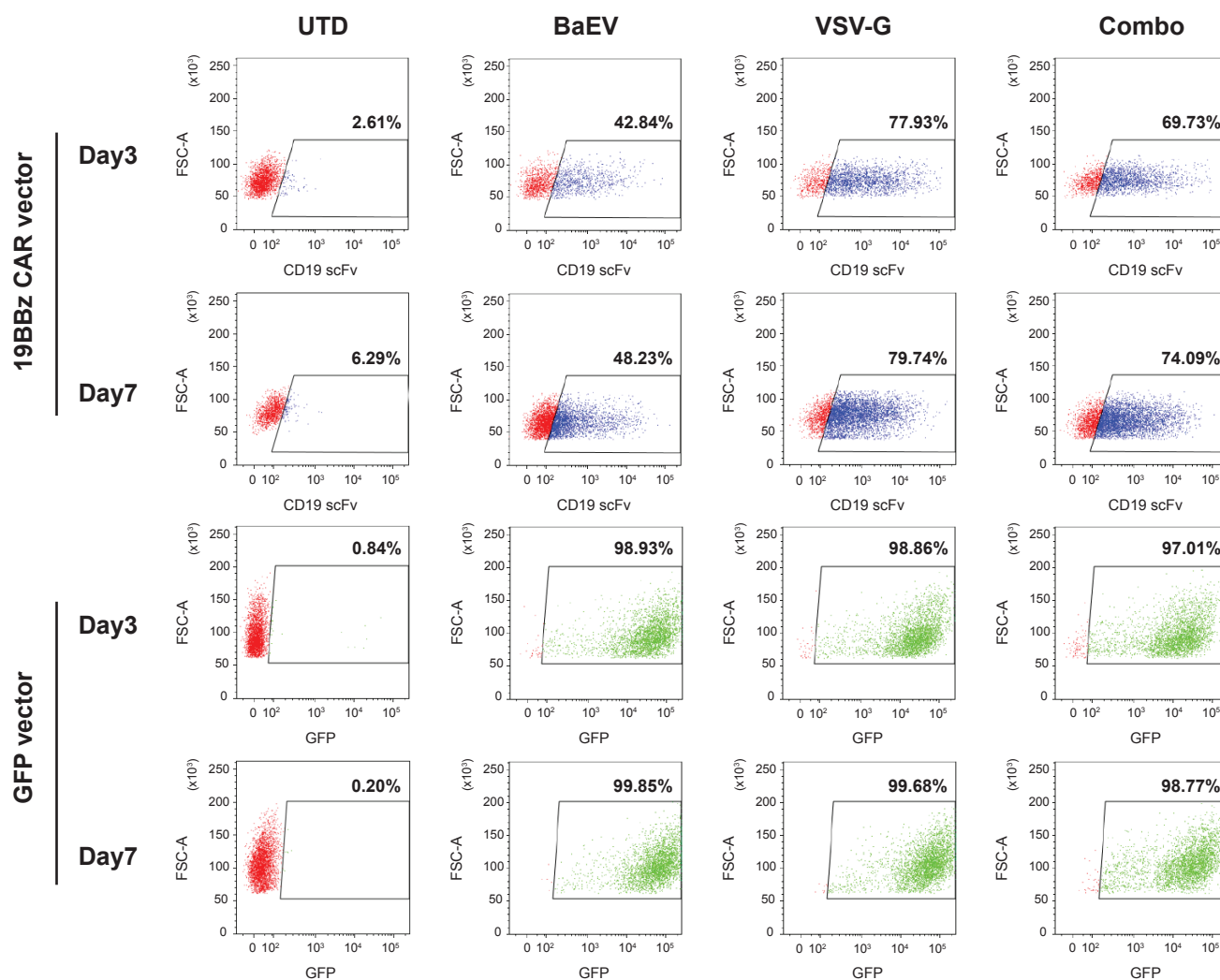

Supplement: S1 Fig — (A) Cell viability evaluated on days 3 and 7 after transduction. The percentages of live, early apoptotic and dead cells were assessed using annexin-V and P.I. staining (n = 3). (B) HEK-293T cells were transduced with each type of LVs. The CD19 scFv-positive cell percentage (left) and GFP-positive cell percentage (right) of each group was assessed on days 3 and 7 post-transduction (n = 3) using flow cytometry analysis. (C) Representative dot plots of CD19 scFv (up) and GFP (down) expressions. Data are presented as mean ± SD. Statistical analysis was performed using a two-way ANOVA with Tukey’s multiple comparisons test (B): ns, non-significant. (PDF) [file pone.0348674.s001.pdf]

**A**

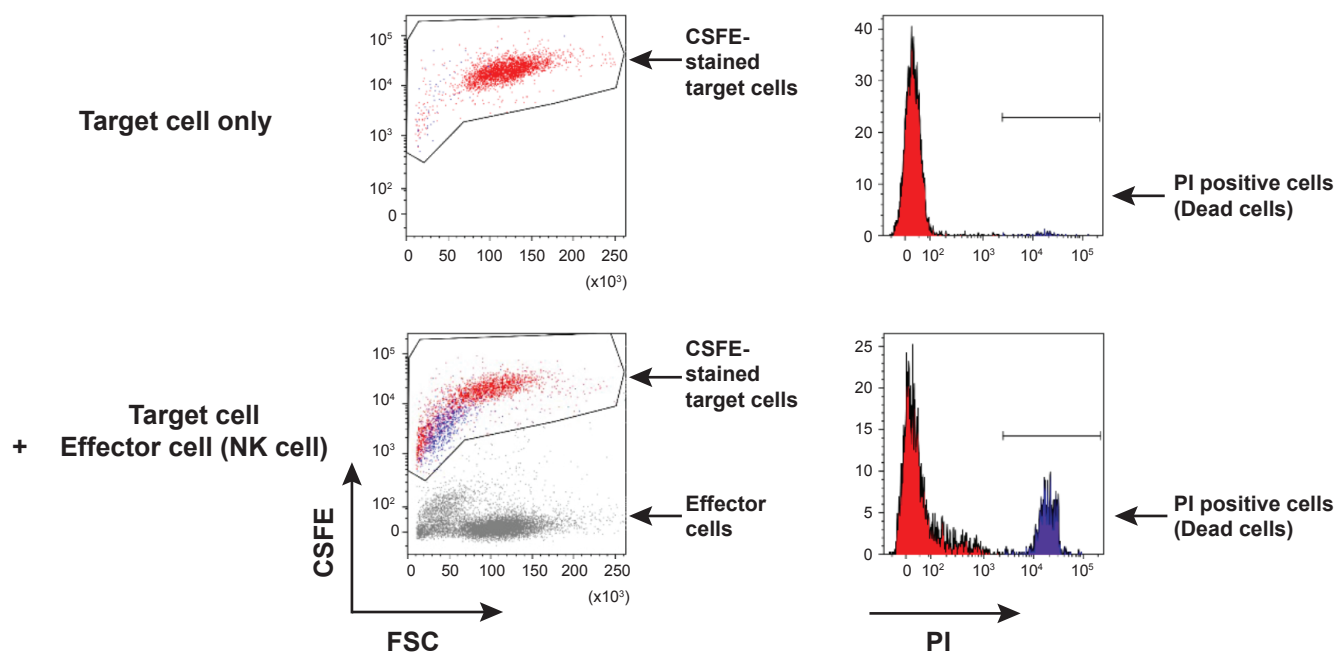

**B**

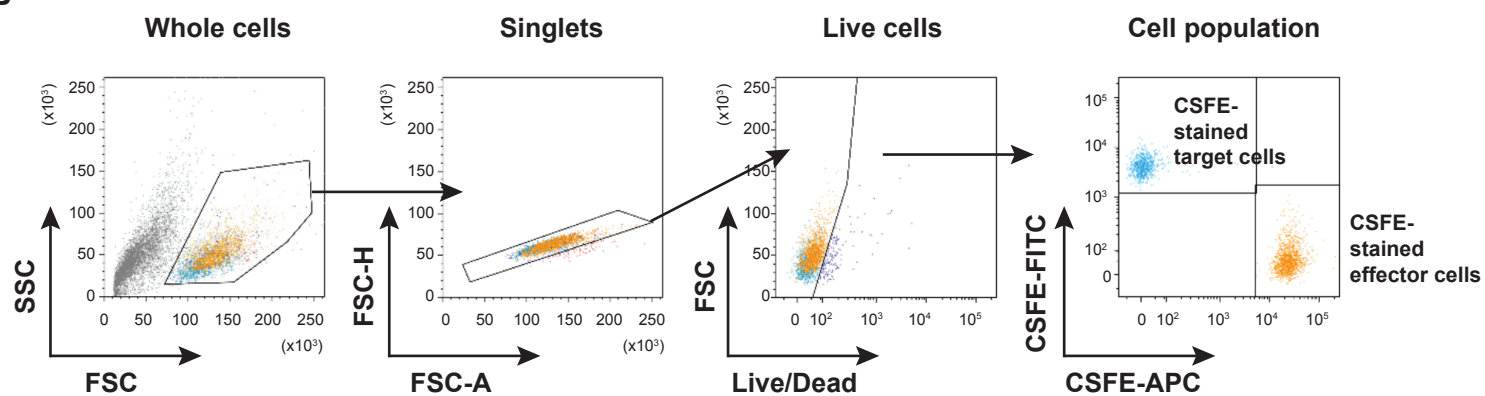

Supplement: S2 Fig — (A, B) Representative gating strategy for (A) short-term killing assay and (B) long-term killing assay. (PDF) [file pone.0348674.s002.pdf]

**A**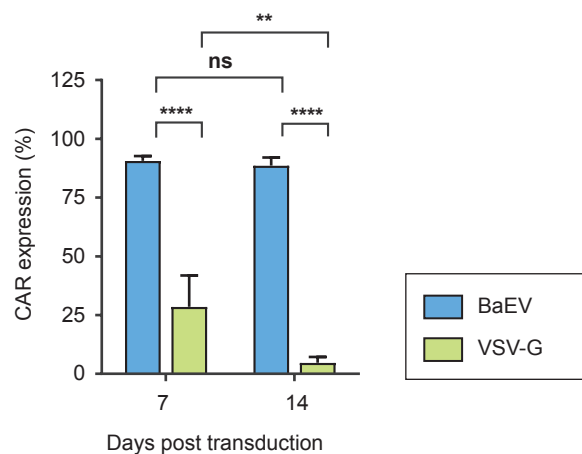**B**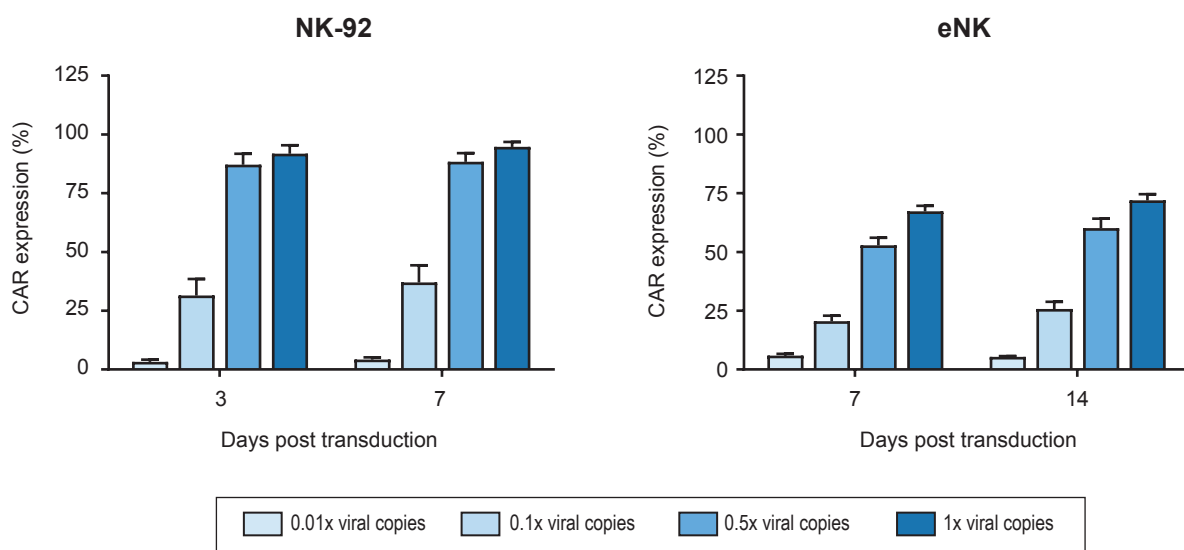

Supplement: S3 Fig — (A) eNK cells were transduced with each type of lentiviral particle. The CAR expression level of each CAR-eNK cell group was assessed by CD19 scFv-positive cell percentage on days 7 and 14 post-transduction (n = 4). (B) CAR expression by NK92 cells and eNK cells transduced with 19BBz CAR using BaEV-LV at indicated viral copy numbers (NK92, n = 3; eNK, n = 7), (0.01x viral copies, 2.32x10^7^; 0.1x viral copies, 2.32x10^8^; 0.5x viral copies, 1.16x10^9^; 1x viral copies, 2.32x10^9^; transduced into a total of 2.5x10^5^ cells). Data are presented as mean ± SD. Statistical analysis was performed using a two-way ANOVA with Tukey’s multiple comparisons test (A): **, p < 0.01; ****, p < 0.0001; ns, non-significant. (PDF) [file pone.0348674.s003.pdf]

**F**

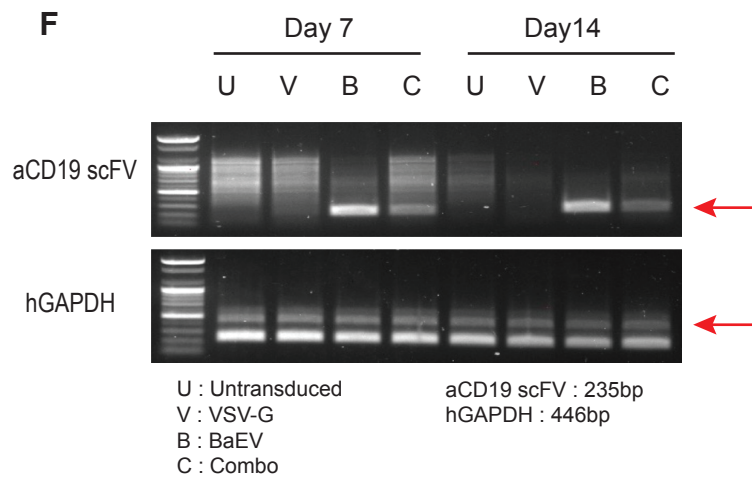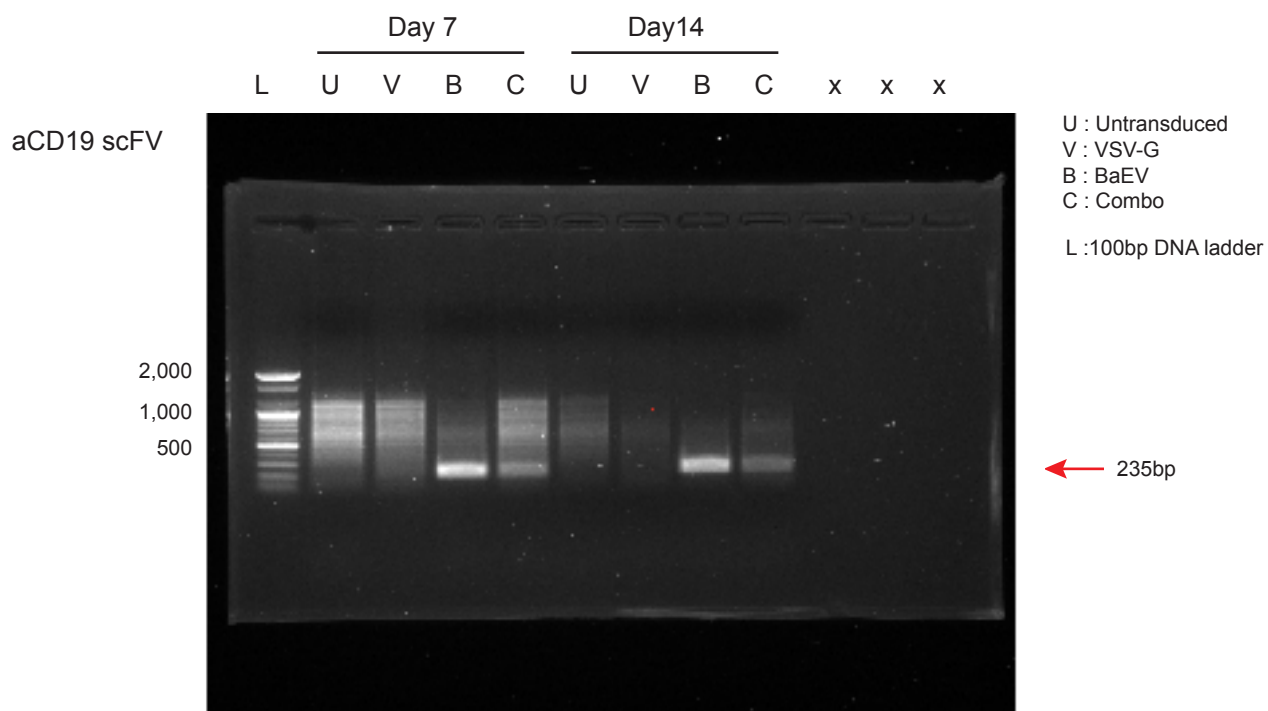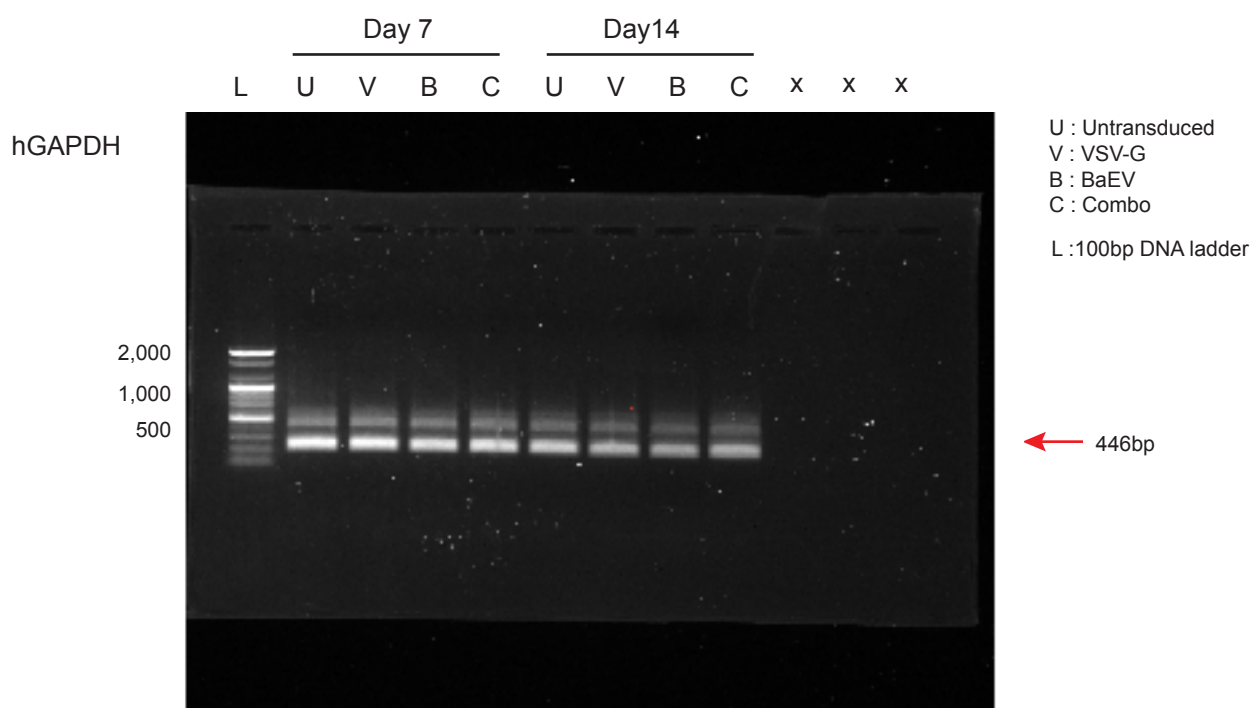

Supplement: S4 Fig — (PDF) [file pone.0348674.s004.pdf]
